# Supplementary material for: Establishment of Mucoepidermoid Carcinoma Cell Lines from Surgical and Recurrence Biopsy Specimens
Source: Int J Mol Sci. 2023 Jan 15;24(2):1722. doi: 10.3390/ijms24021722 (PMC9865506; doi:10.3390/ijms24021722)
Supplement: Supplementary file 1 [file ijms-24-01722-s001.zip › 221007_Supplementary_materials (2).pdf]

SUPPLEMENTARY ONLINE MATERIAL

Materials and Methods

Fluorescent in situ hybridization (FISH)

To prepare cell-culture samples for FISH, cells were propagated under exponential growth condition, dissociated with trypsin, incubated in 40 mM KCl for 15 min at 37 °C, fixed with a combination of 3:1 methanol and glacial acetic acid, and then placed onto glass slides. Slides were hybridized with fluorescently labeled BAC probes. BAC DNA FISH was conducted using probes according to a previously established method [34]. The BAC DNA partially purified with standard alkaline-lysis procedures was labeled with Red 580 dUTP and Green 496 dUTP -Nick Translation Kit (Vysis, Abbot Laboratories, Abbott Park) using DNA polymerase/DNase I (Invitrogen). Denatured slides and probes were hybridized overnight in a humid chamber at 37 °C. The DAPI (4,6-diaminino-2-phenylindole) stained slides were examined using a BX-60-RF fluorescence microscope (Olympus). Image were captured using IP Lab Scientific Imaging Software (Scanalytics).

Exome-sequencing

Repertoire Genesis Inc. (Osaka, Japan) was approached to conduct the sequencing and data processing. Quality control (QC) in exome sequencing library preparation was accomplished through preliminary gDNA quantification on agarose gel electrophoresis using Qubit 2.0. Following the QC processes, 1 μg gDNA per sample was used as the starting material for the DNA sample preparations. Sequencing libraries were generated using the Agilent SureSelect Human All ExonV6 kit (Agilent Technologies, CA, USA) following the manufacturer's recommendations, and index codes were added to attribute sequences to each sample. Hydrodynamic shearing (Covaris, Massachusetts, USA) generated 180-280bp fragments. The combination of exonuclease and polymerase activities, followed by enzyme removal, converted any residual overhangs into blunt ends. Adapter oligonucleotides were ligated after 3' DNA fragment adenylation. PCR selectively enriched adapter-ligated DNA fragments. The PCR reaction was used to enrich captured libraries with index tags prior to hybridization. The products were purified using the AMPure XP system (Beckman Coulter, Beverly, USA) and quantified using the

Agilent high sensitivity DNA assay on the Agilent Bioanalyzer 2100 system. The qualifying libraries were sequenced on an Illumina Novaseq6000 to obtain 150 paired-end sequencing data per sample.

Data processing

Paired-end reads from normal, and tumor exome sequencing were used as raw data. First, adapter fragments and low-quality regions with 10-base moving average QV scores below 12 were removed from both ends of each read, and the resulting read sets were used in subsequent steps. The exome reads were mapped to the human reference genome (hg19) using paired-end BWA-MEM mode [35]. . Somatic mutations (SNVs and short indels) in the tumor genome were identified by comparing the read map of normal and tumor tissues using muTect [36] , VarScan [37], and lofreq [38]. Subsequently, functional annotations were assigned to the mutations using ANNOVAR [39]

Results

FISH analysis for t(11:19) in MEC cells

FISH analysis revealed translocation t(11:19) in the MEC cell lines. The probe was validated with the three cell lines (AMU-MEC1, AMU-MEC1-R1, AMU-MEC1-R2). In each cell, green signals (corresponding to the CRTC1 probe), red signals (corresponding to the MAML2 probe), and yellow or touching signals (corresponding to translocation) were observed (Supplementary Figure S4). One, three, and two fusion signals were found in AMU-MEC1(Supplementary Figure S4A), AMU-MEC1-R1(Supplementary Figure S4B), and AMU-MEC1-R2 (Supplementary Figure S4C), respectively, and these cell lines were evaluated as having the t(11:19) pattern.

Tumor mutation burden (TMB) of tumor specimens from initial surgery and biopsy upon recurrence

TMB was calculated as the total number of somatic mutations in the tumor sample per megabyte bases of coding regions (mut/Mb). TMB in the initial surgical and recurrence biopsy specimens were 0.2 and 4.6, respectively.
