# Supplementary material for: Establishment of Mucoepidermoid Carcinoma Cell Lines from Surgical and Recurrence Biopsy Specimens
Source: Int J Mol Sci. 2023 Jan 15;24(2):1722. doi: 10.3390/ijms24021722 (PMC9865506; doi:10.3390/ijms24021722)
Supplement: Supplementary file 1 [file ijms-24-01722-s001.zip › ijms-2042017-supplementary.pdf]

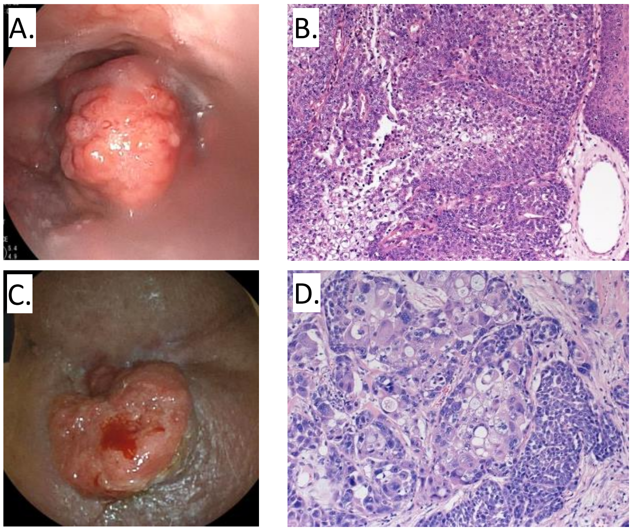

Figure S1. Laryngeal fiberscopy showed a neoplastic lesion bordering the epiglottis. (A) The airway was slightly preserved. (B) HE staining of surgical specimen. (C) A soft lesion tumor appeared just below the tracheal foramen about 2 months after surgery. (D) HE staining of biopsy specimen.

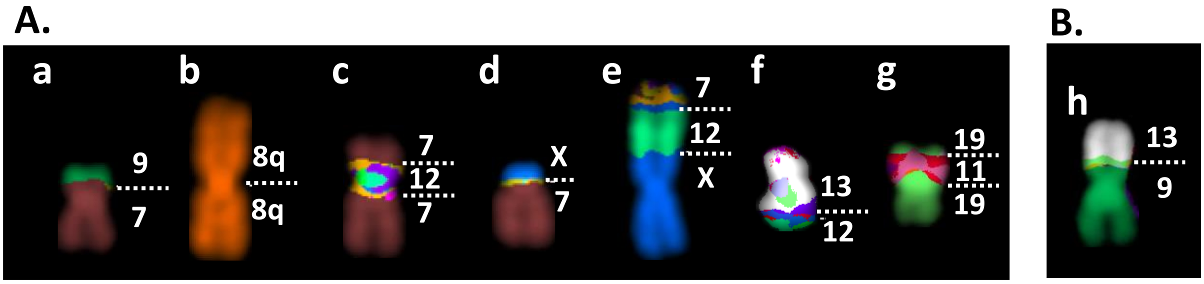

Figure S2. Structural chromosomal abnormalities common to the established MEC cell lines.

(A) Common structural chromosomal abnormalities among the three cell lines. Karyotype of each chromosome is following: a, der(7)t(7;9)(p12;?)del(7)(q21); b, i(8)(q10);
c, der(12)t(7;12)(p11;p11)t(7;12)(q21;q11); d, der(X)t(X;7)(p11;p11)del(X)(q11)+
e, der(X)t(X;12)(p22.1;q13)t(7;12)(?;q24.1); f, der(13)t(12;13)(?;q11);
g, der(19)ins(19;11)(p13;?). (B) Common structural chromosomal abnormality between AMU-MEC1 and AMU-MEC1-R2. Karyotype of this chromosome is der(9)t(9;13)(p?13;q21)

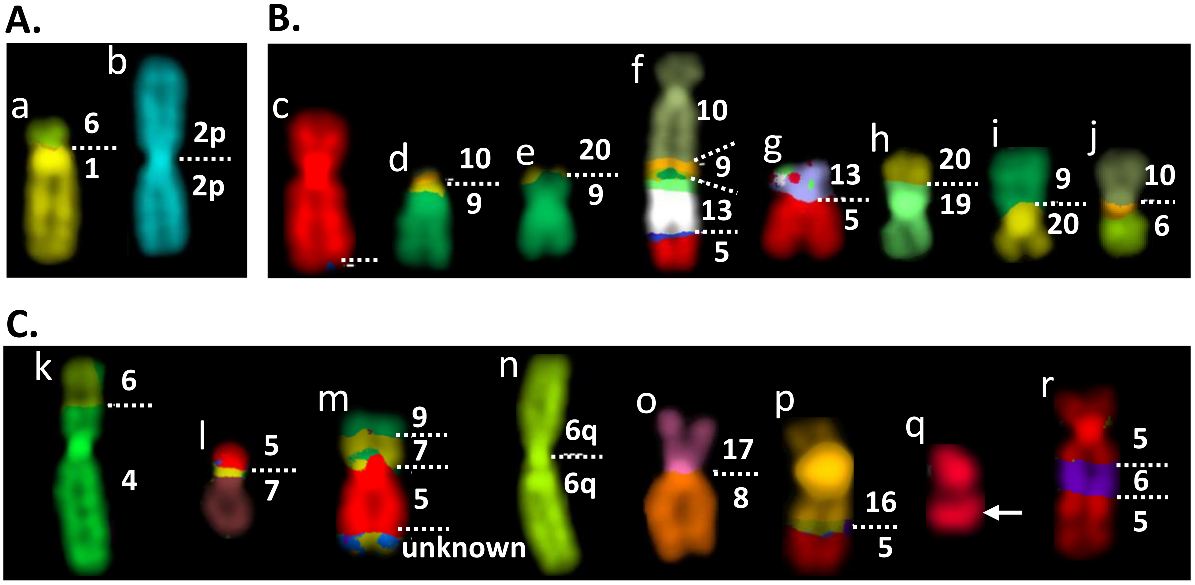

Figure S3. Unique chromosomal abnormalities in each established MEC cell lines. (A) Unique structural chromosomal abnormalities in AMU-MEC1. a, der(1)t(1;6)(p11;?); b, i(2)(p10). (B) Unique structural chromosomal abnormalities in AMU-MEC1-R1. c, del(5)(q31); d, +der(10)del(10)(p11)t(9;10)(q11;q11); e, der(9)t(9;20)(p13;?)del(9)(q21); f, der(10)t(9;10)(?;q25)t(9;13)(?;q3)t(5;13)(q31;q14); g, der(13)t(5;13)(q23;q11~12); h, der(19)t(19;20)(p13;?); i, der(9)t(9;20)(p13;?)del(9)(q21); j, t(6;10)(p21;q11). (C) Unique structural chromosomal abnormalities in AMU-MEC1-R2. k, der(4)t(4;6)(p15;p12); l, der(5)del(5)(p13)t(5;7)(q11;p1?4); m, +der(5)t(5;7)(p1?2;?)t(7;9)(?;?)add(5)(q2?3); n, i(6)(q10); o, der(8;17)(q10;q10); p, der(16)t(5;16)(q23;q21); q, ?dup(22)(q12q13); r, der(5)ins(5;6)(q13;?).

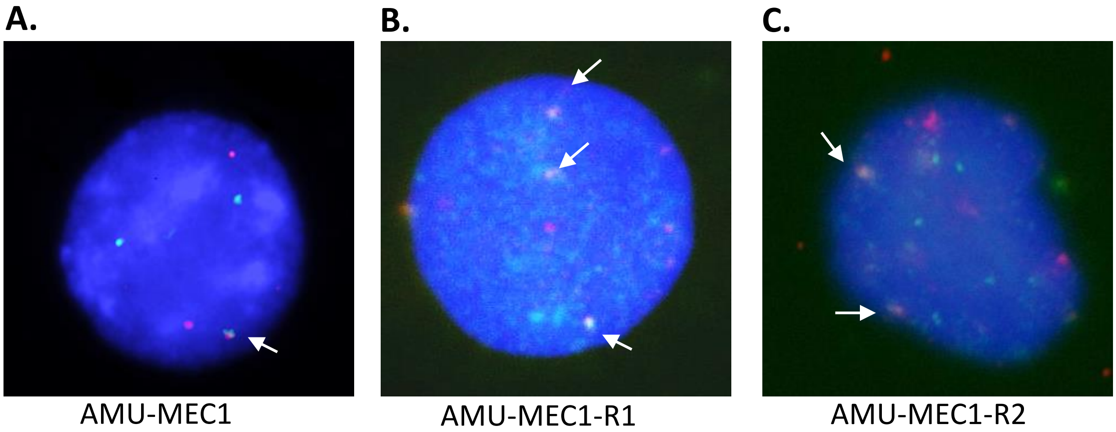

Figure S4. Detection of t(11;19) (q21;p13.2) by FISH in MEC cells. The red signal represents the MAML2 gene and the green CRTC1 gene, while yellow or touching signals indicate fusion of MAML2 and CETC1 genes. White arrows indicate representative fusion signals. (a) A representative case of AMU-MEC1 with t(11;19). (b) b AMU-MEC1-R1 with t(11;19). (c)A AMU-MEC1-R2 with t(11;19).

Supplementary Table S1. Karyotypes in 8 cells of AMU-MEC1

Cell no.	Karyotypes
1	87,Y,+der(X)del(X)(p11)t(X;7)(q11;p11) or +der(X)t(X;7)(p11;p11)del(X)(q11),der(X)t(X;12)(p22.1;q13)t(7;12)(?;q24.1)×2,-Y,-1,+der(1)t(1;6)(p11;?)×2,i(2)(p10),-3,-4,+5,-6,der(6)t(6;6)(p12;q14~16),-7,der(7)t(7;9)(p12;?)del(7)(q21)×3,-8,i(8)(q10),-9,der(9)t(9;13)(p?13;q21),-10,-11,del(11)(q14q22)×2,der(11)t(11;11)(p15;q12),-12,+der(12)t(7;12)(p11;p11)t(7;12)(q21;q11)×4 or +der(12)t(7;12)(q21;p11)t(7;12)(p11;q11)×4,der(12)t(7;12)(p11;p11)t(7;12)(q21;q11)t(7;7)(q36;?) or der(12)t(7;12)(q21;p11)t(7;7)(q36;?)t(7;12)(p11;q11),-13,der(13)t(12;13)(?;q11),-16,der(19)ins(19;11)(p13;?)×2
2	86,Y,+der(X)del(X)(p11)t(X;7)(q11;p11) or +der(X)t(X;7)(p11;p11)del(X)(q11),der(X)t(X;12)(p22.1;q13)t(7;12)(?;q24.1)×2,-Y,-1,+der(1)t(1;6)(p11;?)×2,i(2)(p10),-3,-4,-6,-7,-7,der(7)t(7;9)(p12;?)del(7)(q21)×2,-8,i(8)(q10),-9,der(9)t(9;13)(p?13;q21),-10,del(11)(q14q22)×3,der(11)t(11;11)(p15;q12),-12,-12,+der(12)t(7;12)(p11;p11)t(7;12)(q21;q11)×6 or +der(12)t(7;12)(q21;p11)t(7;12)(p11;q11)×6,-13,der(13)t(12;13)(?;q11),-16,der(19)ins(19;11)(p13;?)×2,-20
3	83,Y,+der(X)del(X)(p11)t(X;7)(q11;p11) or +der(X)t(X;7)(p11;p11)del(X)(q11),der(X)t(X;12)(p22.1;q13)t(7;12)(?;q24.1)×2,-Y,-1,+der(1)t(1;6)(p11;?)×2,i(2)(p10),-3,-4,-4,-6,-7,der(7)t(7;9)(p12;?)del(7)(q21)×3,-8,i(8)(q10),-9,der(9)t(9;13)(p?13;q21),-10,-11,del(11)(q14q22)×2,der(11)t(11;11)(p15;q12),-12,-12,+der(12)t(7;12)(p11;p11)t(7;12)(q21;q11)×5 or +der(12)t(7;12)(q21;p11)t(7;12)(p11;q11)×5,-13,der(13)t(12;13)(?;q11),-16,der(19)ins(19;11)(p13;?)×2,-21,-22
4	83,Y,+der(X)del(X)(p11)t(X;7)(q11;p11) or +der(X)t(X;7)(p11;p11)del(X)(q11),der(X)t(X;12)(p22.1;q13)t(7;12)(?;q24.1)×2,-Y,-1,+der(1)t(1;6)(p11;?)×2,i(2)(p10),-3,-4,-5,-6,-7,-7,der(7)t(7;9)(p12;?)del(7)(q21)×2,-8,i(8)(q10),-9,der(9)t(9;13)(p?13;q21),-10,-11,del(11)(q14q22)×2,der(11)t(11;11)(p15;q12),-12,-12,+der(12)t(7;12)(p11;p11)t(7;12)(q21;q11)×5 or +der(12)t(7;12)(q21;p11)t(7;12)(p11;q11)×5,-13,-13,-16,der(19)ins(19;11)(p13;?)×2
5	85,Y,+der(X)del(X)(p11)t(X;7)(q11;p11) or +der(X)t(X;7)(p11;p11)del(X)(q11),der(X)t(X;12)(p22.1;q13)t(7;12)(?;q24.1)×2,-Y,-1,+der(1)t(1;6)(p11;?)×2,+i(2)(p10),-3,-4,-5,-6,-7,der(7)t(7;9)(p12;?)del(7)(q21)×3,-8,i(8)(q10),-9,der(9)t(9;13)(p?13;q21),-10,-11,del(11)(q14q22)×2,der(11)t(11;11)(p15;q12),-12,-12,+der(12)t(7;12)(p11;p11)t(7;12)(q21;q11)×4 or +der(12)t(7;12)(q21;p11)t(7;12)(p11;q11)×4,-13,der(13)t(12;13)(?;q11),-16,+18,der(19)ins(19;11)(p13;?)×2,-22
6	84,Y,+der(X)del(X)(p11)t(X;7)(q11;p11) or +der(X)t(X;7)(p11;p11)del(X)(q11),der(X)t(X;12)(p22.1;q13)t(7;12)(?;q24.1)×2,-Y,-1,+der(1)t(1;6)(p11;?)×2,i(2)(p10),-3,-4,-5,-6,-7,-7,der(7)t(7;9)(p12;?)del(7)(q21)×2,-8,i(8)(q10),-9,der(9)t(9;13)(p?13;q21),-10,-11,del(11)(q14q22)×2,der(11)t(11;11)(p15;q12),-12,-12,+der(12)t(7;12)(p11;p11)t(7;12)(q21;q11)×5 or +der(12)t(7;12)(q21;p11)t(7;12)(p11;q11)×5,-13,der(13)t(12;13)(?;q11),-16,der(19)ins(19;11)(p13;?)×2
7	84,Y,+der(X)del(X)(p11)t(X;7)(q11;p11) or +der(X)t(X;7)(p11;p11)del(X)(q11),der(X)t(X;12)(p22.1;q13)t(7;12)(?;q24.1)×2,-Y,-1,+der(1)t(1;6)(p11;?)×2,i(2)(p10),-3,-4,-5,-6,-7,-7,der(7)t(7;9)(p12;?)del(7)(q21)×2,-8,i(8)(q10),-9,der(9)t(9;13)(p?13;q21),-10,-11,del(11)(q14q22)×2,der(11)t(11;11)(p15;q12),-12,-12,+der(12)t(7;12)(p11;p11)t(7;12)(q21;q11)×5 or +der(12)t(7;12)(q21;p11)t(7;12)(p11;q11)×5,-13,der(13)t(12;13)(?;q11),-16,der(19)ins(19;11)(p13;?)×2
8	84,Y,+der(X)del(X)(p11)t(X;7)(q11;p11) or +der(X)t(X;7)(p11;p11)del(X)(q11),der(X)t(X;12)(p22.1;q13)t(7;12)(?;q24.1)×2,-Y,-1,+der(1)t(1;6)(p11;?)×2,i(2)(p10),-3,-4,dup(4)(q21q31),-5,-6,-7,-7,der(7)t(7;9)(p12;?)del(7)(q21)×2,-8,i(8)(q10),-9,der(9)t(9;13)(p?13;q21),-10,-11,del(11)(q14q22)×2,der(11)t(11;11)(p15;q12),-12,-12,+der(12)t(7;12)(p11;p11)t(7;12)(q21;q11)×5 or +der(12)t(7;12)(q21;p11)t(7;12)(p11;q11)×5,-13,der(13)t(12;13)(?;q11),-16,der(19)ins(19;11)(p13;?)×2

Supplementary Table S2. Karyotypes in 8 cells of AMU-MEC1-R1

Cell no.	Karyotypes
**1**	48,Y,+der(X)del(X)(p11)t(X;7)(q11;p11) or +der(X)t(X;7)(p11;p11)del(X)(q11),der(X)t(X;12)(p22.1;q13)t(7;12)(?;q24.1),del(5)(q31),der(7)t(7;9)(p12;?)del(7)(q21)×2,+i(8)(q10),-9,der(9)t(9;20)(p13;?)del(9)(q21),+der(10)del(10)(p11)t(9;10)(q11;q11) or der(10)t(9;10)(q11;p11)del(10)(q11),der(10)t(9;10)(?;q25)t(9;13)(?;q3)t(5;13)(q31;q14),del(11)(q14q22),der(12)t(7;12)(p11;p11)t(7;12)(q21;q11) or der(12)t(7;12)(q21;p11)t(7;12)(p11;q11),der(13)t(5;13)(q23;q11~12),der(19)ins(19;11)(p13;?),der(19)t(19;20)(p13;?),der(20)t(9;20)(?;p11)
**2**	48,Y,+der(X)del(X)(p11)t(X;7)(q11;p11) or +der(X)t(X;7)(p11;p11)del(X)(q11),der(X)t(X;12)(p22.1;q13)t(7;12)(?;q24.1),del(5)(q31),der(7)t(7;9)(p12;?)del(7)(q21)×2,+i(8)(q10),-9,der(9)t(9;20)(p13;?)del(9)(q21),+der(10)del(10)(p11)t(9;10)(q11;q11) or der(10)t(9;10)(q11;p11)del(10)(q11),der(10)t(9;10)(?;q25)t(9;13)(?;q3)t(5;13)(q31;q14),del(11)(q14q22),der(12)t(7;12)(p11;p11)t(7;12)(q21;q11) or der(12)t(7;12)(q21;p11)t(7;12)(p11;q11),der(13)t(5;13)(q23;q11~12),der(19)ins(19;11)(p13;?),der(19)t(19;20)(p13;?),der(20)t(9;20)(?;p11)
**3**	48,Y,+der(X)del(X)(p11)t(X;7)(q11;p11) or +der(X)t(X;7)(p11;p11)del(X)(q11),der(X)t(X;12)(p22.1;q13)t(7;12)(?;q24.1),del(5)(q31),t(6;10)(p21;q11),der(7)t(7;9)(p12;?)del(7)(q21)×2,+der(8)t(4;8)(?;q24),i(8)(q10),-9,der(9)t(9;20)(p13;?)del(9)(q21),+der(10)del(10)(p11)t(9;10)(q11;q11) or der(10)t(9;10)(q11;p11)del(10)(q11),der(10)?dup(10)(p15p11.2)t(9;10)(?;q25)t(9;13)(?;q3)t(5;13)(q31;q14),del(11)(q14q22),der(12)t(7;12)(p11;p11)t(7;12)(q21;q11) or der(12)t(7;12)(q21;p11)t(7;12)(p11;q11),i(13)(q10),der(13)t(12;13)(?;q11),der(19)ins(19;11)(p13;?),der(19)t(19;20)(p13;?),der(20)t(9;20)(?;p11)
**4**	48,Y,+der(X)del(X)(p11)t(X;7)(q11;p11) or +der(X)t(X;7)(p11;p11)del(X)(q11),der(X)t(X;12)(p22.1;q13)t(7;12)(?;q24.1),del(5)(q31),der(7)t(7;9)(p12;?)del(7)(q21)×2,+i(8)(q10),-9,der(9)t(9;20)(p13;?)del(9)(q21),+der(10)del(10)(p11)t(9;10)(q11;q11) or der(10)t(9;10)(q11;p11)del(10)(q11),der(10)t(9;10)(?;q25)t(9;13)(?;q3)t(5;13)(q31;q14),del(11)(q14q22),der(12)t(7;12)(p11;p11)t(7;12)(q21;q11) or der(12)t(7;12)(q21;p11)t(7;12)(p11;q11),der(13)t(5;13)(q23;q11~12),der(19)ins(19;11)(p13;?),der(19)t(19;20)(p13;?),der(20)t(9;20)(?;p11)
**5**	48,Y,+der(X)del(X)(p11)t(X;7)(q11;p11) or +der(X)t(X;7)(p11;p11)del(X)(q11),der(X)t(X;12)(p22.1;q13)t(7;12)(?;q24.1),del(2)(p2?1),del(5)(q31),der(7)t(7;9)(p12;?)del(7)(q21)×2,+i(8)(q10),-9,der(9)t(9;20)(p13;?)del(9)(q21),+der(10)del(10)(p11)t(9;10)(q11;q11) or der(10)t(9;10)(q11;p11)del(10)(q11),der(10)t(9;10)(?;q25)t(9;13)(?;q3)t(5;13)(q31;q14),del(11)(q14q22),der(12)t(7;12)(p11;p11)t(7;12)(q21;q11) or der(12)t(7;12)(q21;p11)t(7;12)(p11;q11),der(13)t(5;13)(q23;q11~12),der(19)ins(19;11)(p13;?),der(19)t(19;20)(p13;?),der(20)t(9;20)(?;p11)
**6**	47,Y,+der(X)del(X)(p11)t(X;7)(q11;p11) or +der(X)t(X;7)(p11;p11)del(X)(q11),der(X)t(X;12)(p22.1;q13)t(7;12)(?;q24.1),dic(1;22)(p36.3;p11),del(5)(q31),der(7)t(7;9)(p12;?)del(7)(q21)×2,+i(8)(q10),-9,der(9)t(9;20)(p13;?)del(9)(q21),+der(10)del(10)(p11)t(9;10)(q11;q11) or der(10)t(9;10)(q11;p11)del(10)(q11),der(10)t(9;10)(?;q25)t(9;13)(?;q3)t(5;13)(q31;q14),del(11)(q14q22),der(12)t(7;12)(p11;p11)t(7;12)(q21;q11) or der(12)t(7;12)(q21;p11)t(7;12)(p11;q11),der(13)t(12;13)(?;q11),der(18)t(13;18)(q3;q22)t(5;13)(q31;q14),der(19)ins(19;11)(p13;?),der(19)t(19;20)(p13;?),der(20)t(9;20)(?;p11)
**7**	48,Y,+der(X)del(X)(p11)t(X;7)(q11;p11) or +der(X)t(X;7)(p11;p11)del(X)(q11),der(X)t(X;12)(p22.1;q13)t(7;12)(?;q24.1),del(2)(p11.1),del(5)(q31),der(7)t(7;9)(p12;?)del(7)(q21)×2,+i(8)(q10),-9,der(9)t(9;20)(p13;?)del(9)(q21),+der(10)del(10)(p11)t(9;10)(q11;q11) or der(10)t(9;10)(q11;p11)del(10)(q11),der(10)t(9;10)(?;q25)t(9;13)(?;q3)t(5;13)(q31;q14),del(11)(q14q22),der(12)t(7;12)(p11;p11)t(7;12)(q21;q11) or der(12)t(7;12)(q21;p11)t(7;12)(p11;q11),der(13)t(5;13)(q23;q11~12),der(19)ins(19;11)(p13;?),der(19)t(19;20)(p13;?),der(20)t(9;20)(?;p11)
**8**	51,Y,+der(X)del(X)(p11)t(X;7)(q11;p11) or +der(X)t(X;7)(p11;p11)del(X)(q11),der(X)t(X;12)(p22.1;q13)t(7;12)(?;q24.1),+del(5)(q14~15),del(5)(q31),t(6;10)(p21;q11),der(7)t(7;9)(p12;?)del(7)(q21)×2,+i(8)(q10)×2,-9,der(9)t(9;20)(p13;?)del(9)(q21),+der(10)del(10)(p11)t(9;10)(q11;q11) or der(10)t(9;10)(q11;p11)del(10)(q11),der(10)t(9;10)(?;q25)t(9;13)(?;q3)t(5;13)(q31;q14),+del(11)(q14q22),der(12)t(7;12)(p11;p11)t(7;12)(q21;q11) or der(12)t(7;12)(q21;p11)t(7;12)(p11;q11),der(13)t(12;13)(?;q11),der(19)ins(19;11)(p13;?),der(19)t(19;20)(p13;?),der(20)t(9;20)(?;p11)

Supplementary Table S3. Karyotypes in 8 cells of AMU-MEC1-R2

Cell no.	Karyotypes
1	89,YY,+der(X)del(X)(p11)t(X;7)(q11;p11)×2 or +der(X)t(X;7)(p11;p11)del(X)(q11)×2,der(X)t(X;12)(p22.1;q13)t(7;12)(?;q24.1)×2,-1,del(2)(q22),4,der(4)t(4;6)(p15;p12),der(5)del(5)(p13)t(5;7)(q11;p1?4)×2,der(5)ins(5;6)(q13;?),+der(5)t(5;7)(p1?2;?)t(7;9)(?;?)add(5)(q2?3),i(6)(q10)×2,-7,-7,der(7)t(7;9)(p12;?)del(7)(q21)×2,+der(8;17)(q10;q10),i(8)(q10)×2,der(9)t(9;13)(p?13;q21)×2,10,del(11)(q14q22)×2,der(12)t(7;12)(p11;p11)t(7;12)(q21;q11)×2 or der(12)t(7;12)(q21;p11)t(7;12)(p11;q11)×2,der(13)t(12;13)(?;q11)×2,der(16)t(5;16)(q23;q21)×2,-17,-17,der(19)ins(19;11)(p13;?)×2,?dup(22)(q12q13)
2	85,YY,+der(X)del(X)(p11)t(X;7)(q11;p11) or +der(X)t(X;7)(p11;p11)del(X)(q11),der(X)t(X;12)(p22.1;q13)t(7;12)(?;q24.1)×2,-1,-2,-4,der(4)t(4;6)(p15;p12),der(5)del(5)(p13)t(5;7)(q11;p1?4)×2,der(5)ins(5;6)(q13;?),+der(5)t(5;7)(p1?2;?)t(7;9)(?;?)add(5)(q2?3),-6,der(6)t(?X;6)(?;p21),-7,-7,der(7)t(7;9)(p12;?)del(7)(q21)×2,der(8;17)(q10;q10),i(8)(q10),der(9)t(9;13)(p?13;q21)×2,-10,del(11)(q14q22)×2,der(12)t(7;12)(p11;p11)t(7;12)(q21;q11)×2 or der(12)t(7;12)(q21;p11)t(7;12)(p11;q11)×2,der(13)t(12;13)(?;q11)×2,der(16)t(5;16)(q23;q21)×2,-17,-18,der(19)ins(19;11)(p13;?)×2,+20,-21,+22,der(22)t(18;22)(q?;p11)×2
3	89,YY,+der(X)del(X)(p11)t(X;7)(q11;p11)×2 or +der(X)t(X;7)(p11;p11)del(X)(q11)×2,der(X)t(X;12)(p22.1;q13)t(7;12)(?;q24.1)×2,-1,-4,der(4)t(4;6)(p15;p12),der(5)del(5)(p13)t(5;7)(q11;p1?4)×2,+der(5)ins(5;6)(q13;?),+der(5)t(5;7)(p1?2;?)t(7;9)(?;?)add(5)(q2?3),i(6)(q10)×2,-7,-7,der(7)t(7;9)(p12;?)del(7)(q21)×2,+der(8;17)(q10;q10),i(8)(q10)×2,der(9)t(9;13)(p?13;q21)×2,-10,del(11)(q13),del(11)(q14q22),der(12)t(7;12)(p11;p11)t(7;12)(q21;q11)×2 or der(12)t(7;12)(q21;p11)t(7;12)(p11;q11)×2,-13,der(13)t(12;13)(?;q11),der(16)t(5;16)(q23;q21)×2,-17,-17,der(19)ins(19;11)(p13;?)×2,der(22)t(11;22)(q13;p11)del(11)(q14q22)?dup(22)(q12q13)
4	86,YY,+der(X)del(X)(p11)t(X;7)(q11;p11)×2 or +der(X)t(X;7)(p11;p11)del(X)(q11)×2,der(X)t(X;12)(p22.1;q13)t(7;12)(?;q24.1)×2,-1,-4,der(4)t(4;6)(p15;p12),der(5)del(5)(p13)t(5;7)(q11;p1?4)×2,der(5)ins(5;6)(q13;?),+der(5)t(5;7)(p1?2;?)t(7;9)(?;?)add(5)(q2?3),-6,der(6)t(6;16)(p21;?)t(16;18)(?;q2?2),-7,-7,der(7)t(7;9)(p12;?)del(7)(q21)×2,der(8;17)(q10;q10),i(8)(q10),der(9)t(9;13)(p?13;q21)×2,-10,del(11)(q14q22)×2,der(12)t(7;12)(p11;p11)t(7;12)(q21;q11)×2 or der(12)t(7;12)(q21;p11)t(7;12)(p11;q11)×2,der(13)t(12;13)(?;q11)×2,der(16)t(5;16)(q23;q21)×2,-17,-18,der(19)ins(19;11)(p13;?)×2,der(22)t(X;22)(?;p11)
5	89,YY,+der(X)del(X)(p11)t(X;7)(q11;p11)×2 or +der(X)t(X;7)(p11;p11)del(X)(q11)×2,der(X)t(X;12)(p22.1;q13)t(7;12)(?;q24.1)×2,-1,-4,der(4)t(4;6)(p15;p12),der(5)del(5)(p13)t(5;7)(q11;p1?4)×2,+der(5)ins(5;6)(q13;?),+der(5)t(5;7)(p1?2;?)t(7;9)(?;?)add(5)(q2?3),i(6)(q10)×2,-7,-7,der(7)t(7;9)(p12;?)del(7)(q21)×2,+der(8;17)(q10;q10),i(8)(q10)×2,der(9)t(9;13)(p?13;q21)×2,-10,del(11)(q14q22)×2,der(12)t(7;12)(p11;p11)t(7;12)(q21;q11)×2 or der(12)t(7;12)(q21;p11)t(7;12)(p11;q11)×2,-13,der(13)t(12;13)(?;q11),der(16)t(5;16)(q23;q21)×2,-17,-17,der(19)ins(19;11)(p13;?)×2,?dup(22)(q12q13)
6	86,YY,+der(X)del(X)(p11)t(X;7)(q11;p11) or +der(X)t(X;7)(p11;p11)del(X)(q11),der(X)t(X;12)(p22.1;q13)t(7;12)(?;q24.1)×2,-1,der(2)t(2;21)(q22;?),+3,-4,-4,der(4)t(4;6)(p15;p12),der(5)del(5)(p13)t(5;7)(q11;p1?4)×2,der(5)ins(5;6)(q13;?),+der(5)t(5;7)(p1?2;?)t(7;9)(?;?)add(5)(q2?3),der(6)t(6;14)(q10;q10),i(6)(q10),-7,-7,der(7)t(7;9)(p12;?)del(7)(q21)×2,der(8;11)tas(8;11)(q24;p15)del(11)(q14q22),+der(8;17)(q10;q10),i(8)(q10)×2,der(9)t(9;13)(p?13;q21)×2,del(11)(q14q22),der(12)t(7;12)(p11;p11)t(7;12)(q21;q11)×2 or der(12)t(7;12)(q21;p11)t(7;12)(p11;q11)×2,der(13)t(12;13)(?;q11)×2,der(16)t(5;16)(q23;q21)×2,-17,-17,der(19)ins(19;11)(p13;?)×2,-21,-21,?dup(22)(q12q13)
7	89,YY,+der(X)del(X)(p11)t(X;7)(q11;p11)×2 or +der(X)t(X;7)(p11;p11)del(X)(q11)×2,der(X)t(X;12)(p22.1;q13)t(7;12)(?;q24.1)×2,-1,-4,der(4)t(4;6)(p15;p12),der(5)del(5)(p13)t(5;7)(q11;p1?4)×2,+der(5)ins(5;6)(q13;?),+der(5)t(3;5)(?;q1?2),der(5)t(5;7)(p1?2;?)t(7;9)(?;?)add(5)(q2?3),i(6)(q10)×2,-7,-7,der(7)t(7;9)(p12;?)del(7)(q21)×2,+der(8;17)(q10;q10),i(8)(q10)×2,der(9)t(9;13)(p?13;q21)×2,-10,del(11)(q14q22)×2,der(12)t(7;12)(p11;p11)t(7;12)(q21;q11)×2 or der(12)t(7;12)(q21;p11)t(7;12)(p11;q11)×2,-13,der(13)t(12;13)(?;q11),der(16)t(5;16)(q23;q21)×2,-17,-17,der(19)ins(19;11)(p13;?),der(19)ins(19;11)(p13;?)t(3;19)(?;q11~12),?dup(22)(q12q13)
8	85,YY,+der(X)del(X)(p11)t(X;7)(q11;p11)×2 or +der(X)t(X;7)(p11;p11)del(X)(q11)×2,der(X)t(X;12)(p22.1;q13)t(7;12)(?;q24.1)×2,-Y,-1,-2,-4,der(4)t(4;6)(p15;p12),der(5)del(5)(p13)t(5;7)(q11;p1?4)×2,der(5)ins(5;6)(q13;?),+der(5)t(5;7)(p1?2;?)t(7;9)(?;?)add(5)(q2?3),i(6)(q10)×2,-7,-7,-7,der(7)t(7;9)(p12;?)del(7)(q21),i(8)(q10)×2,der(9)t(9;13)(p?13;q21)×2,-10,del(11)(q14q22)×2,der(12)t(7;12)(p11;p11)t(7;12)(q21;q11)×2 or der(12)t(7;12)(q21;p11)t(7;12)(p11;q11)×2,+der(12)t(7;12)(p11;p11)t(7;12)(q21;q11)dup(7)(q21q36) or +der(12)t(7;12)(q21;p11)dup(7)(q21q36)t(7;12)(p11;q11),-13,der(13)t(12;13)(?;q11),-16,der(16)t(5;16)(q23;q21)×2,-17,-17,+18,der(19)ins(19;11)(p13;?)×2,?dup(22)(q12q13)
